# Supplementary material for: Disorder compensation controls doping efficiency in organic semiconductors
Source: Nat Commun. 2019 Oct 7;10:4547. doi: 10.1038/s41467-019-12526-6 (PMC6779899; doi:10.1038/s41467-019-12526-6)
Supplement: Supplementary file 1 — Supplementary Information [file 41467_2019_12526_MOESM1_ESM.pdf]

Supplementary Information

Disorder compensation controls doping efficiency in organic semiconductors

Fediai *et al.*

Supplementary Figures 3
Supplementary Figure 1 3
Supplementary Figure 2 3
Supplementary Figure 3 4
Supplementary Figure 4 4
Supplementary Figure 5 5
Supplementary Figure 6 5
Supplementary Figure 7 6
Supplementary Figure 8 6
Supplementary Notes 7
Supplementary Note 1: Polaron dynamics in doped organic semiconductors 7
Supplementary Note 2: Microscopic processes 8
Supplementary Note 3: Extraction of the HOMO and $LUMO^+$ distribution 10
Supplementary Note 4: Extraction of the bulk Fermi level shift 11
In case of the "bulk semiconductor" 11
In case of "single layer devices" 12
Supplementary Note 5: The Fermi level shift in the thin doped layers 13
Supplementary Note 6: Disorder correlation for the occupied and free states 13
Supplementary Note 7: Effect of the trap states filling 14
Supplementary References 15

Supplementary Figures

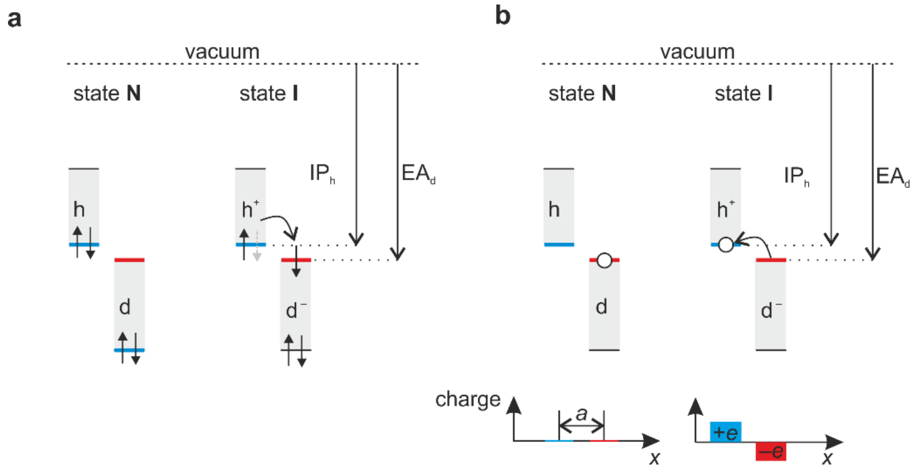

Supplementary Figure 1. Ionization of a dopant molecule in organic semiconductor. a, Frontier molecular orbitals and bonding energies of the neutral (“**N**”) and ionized (“**I**”) states of an isolated infinitely separated host-dopant pair. **b**, The process of the host-dopant pair ionization can be represented as a hop of the positive polaron (hole) from the LUMO of the dopant (“D”) to the HOMO of the host (“H”) shown by the arrow. If the separation of the dopant-host pair (denoted by a) is finite, the Coulomb interaction energy of the generated charge pairs ($+e$ and $-e$ with e being elementary charge) has to be taken into account. Difference in the hole energy and the Coulomb interaction energy are added up to give the energy difference between the ionized and the neutral state – dopant ionization energy.

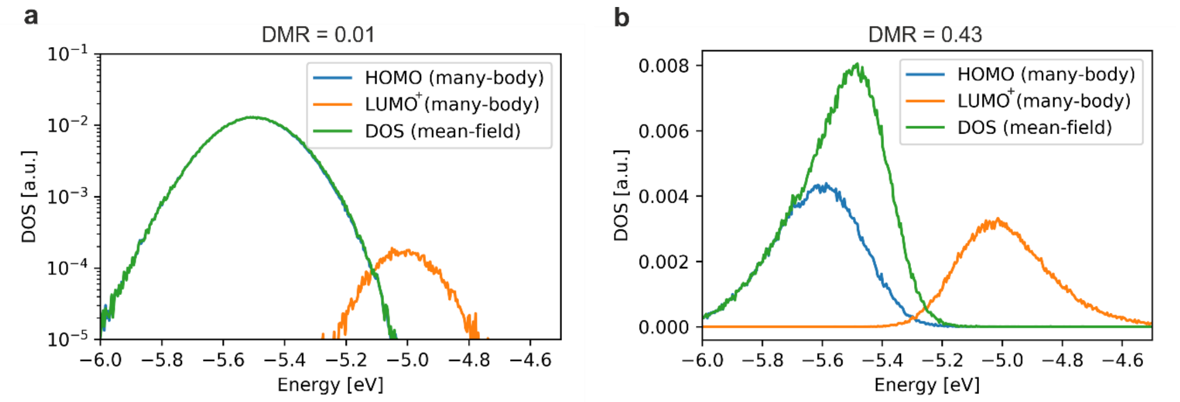

Supplementary Figure 2. Comparison of the two approaches to computing the density of states (DOS). The first approach is referred to as “many-body”, the second approach is referred to as the “mean-field” method. **a**, HOMO and LUMO+ distributions comprising DOS computed according to “many-body” method as well as DOS computed according to the “mean-field” method at dopant molar ratio (DMR) of 0.01. **b**, The same as (a) for DMR of 0.43 (b). Note that throughout this work we used exclusively the “many body” approach that takes into account correlations and as a result predicts LUMO+ dense distribution due to charge-transfer states. Computations were done for a material with 0.1 eV intrinsic disorder and dielectric permittivity $\varepsilon = 4$. Supplementary Note 3 describes implementation of both approaches.

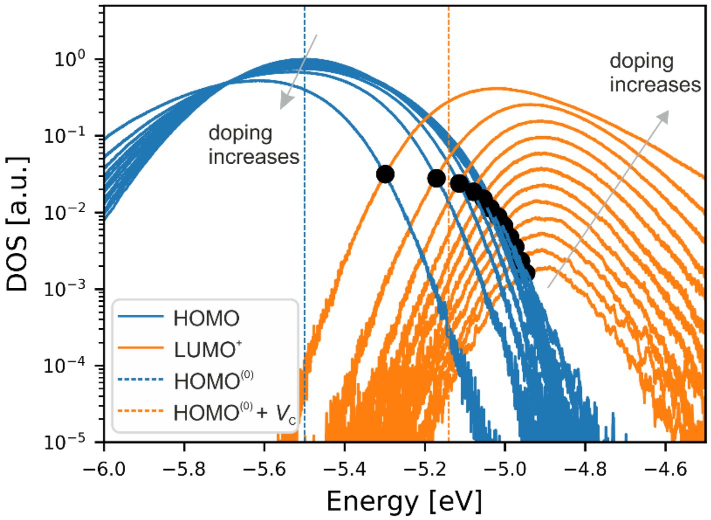

Supplementary Figure 3. HOMO and LUMO⁺ distributions plotted for various dopant molar ratios. The Fermi level as determined from the charge neutrality equation (see Supplementary Note 4 for details) always hits the intersection of the LUMO⁺ and HOMO distribution. This is because holes associated with LUMO distribution and electrons associated with HOMO distribution obey Fermi statistics, in which the Fermi level corresponds to the energy where the state (if exists) is occupied with 50% probability. Therefore, the density of LUMO⁺ and HOMO states is always the same at this energy. Dashed lines show nominal mean value of the HOMO in the corresponding pristine material ($HOMO^{(0)}$) and the expected position of the LUMO⁺ in the weakly doped material with zero disorder ($HOMO^{(0)} + V_C$). HOMO and LUMO+ distributions are computed for bulk material with intrinsic disorder of 0.15 eV and eleven dopant molar ratios between 10^{-3} and 0.4 (dopant molar ratios are logarithmically spaced).

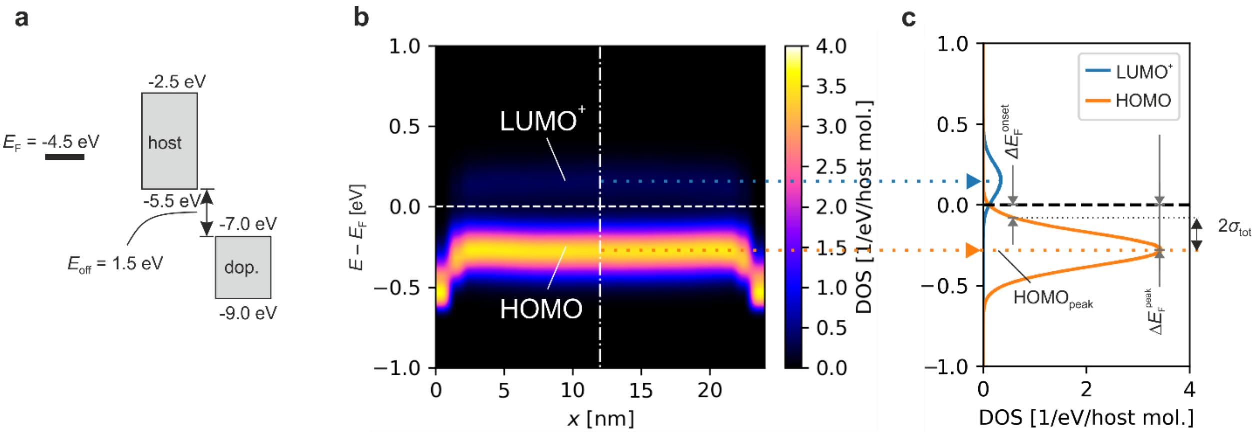

Supplementary Figure 4. Density of states (DOS) in the "single layer device". a, Frontier orbital energy and offset energy of host and guest molecules and the Fermi energy of the electrodes used in simulations. **b**, Computed DOS as a function of the distance x to the left electrode. **c**, Distribution of the LUMO⁺ and HOMO energies in the center of the organic layer. Two definitions of the Fermi level shift, with respect to the peak ($HOMO_{peak}$) and the onset ($HOMO_{onset}$) of the HOMO distribution (ΔE_F^{peak} and ΔE_F^{onset}, respectively) are demonstrated. σ_{tot} stands for the standard deviation of the HOMO distribution (HOMO disorder). The energy E in (b) and (c) is measured with respect to the Fermi level E_F set by electrodes. The dopant molar ratio in these simulations is 8%.

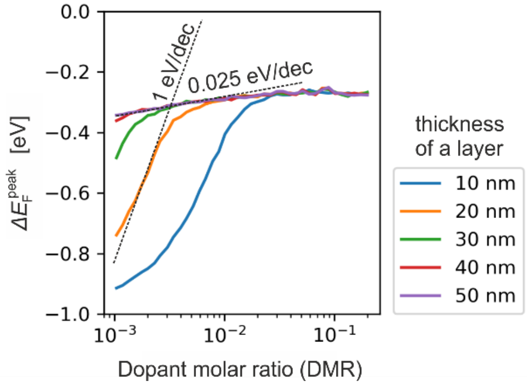

Supplementary Figure 5. Fermi level shift w.r.t. peak of the HOMO (ΔE_F^{peak}) in the center of the doped semiconductor layer sandwiched between two electrodes. Simulations are done for different thickness of the layer. As the doped layer becomes thicker, the position of the "turning point" between the steep (1 eV per decade) and flat (0.025 eV per decade) regions shifts towards smaller molar ratios, demonstrating that the origin of the steep region in the dependence of the Fermi level vs. dopant molar ratio is an interface, not a bulk effect.

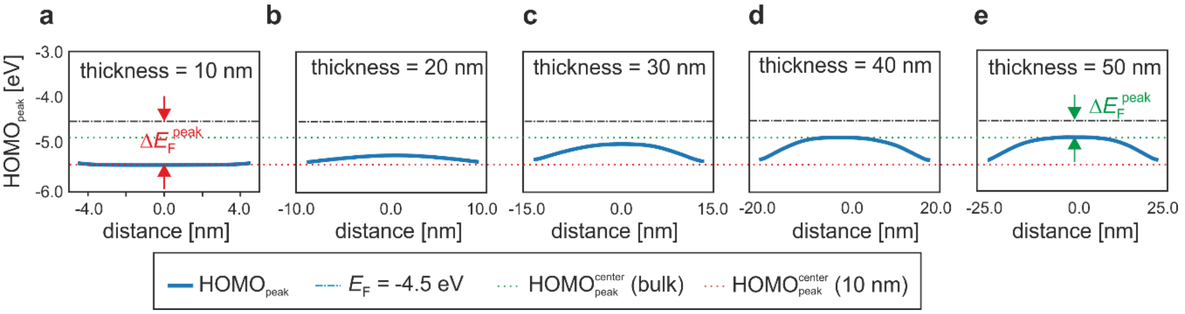

Supplementary Figure 6. The evolution of the HOMO peak depending on the layer thickness. a–e , Peak of the HOMO distribution ($HOMO_{peak}$) as a function of the distance to the center of the "single-layer device" at five different layer thicknesses from 10 nm **(a)** to 50 nm **(e)**. Dopant molar ratio is 0.1%. E_F denotes Fermi level, bulk value of the HOMO peak and the HOMO peak in the center of the smallest, 10 nm sample are denoted as $HOMO_{peak}^{center}$ (bulk) and $HOMO_{peak}^{center}$ (10 nm), respectively. Shift of the Fermi level with respect to the HOMO peak is denoted by ΔE_F^{peak}. For small thickness, the HOMO peak in the center of the simulated layer may look flat, although an organic film is fully depleted. Only for the thickness of about 40...50 nm (see (d) and (e)), the HOMO in the center of the system stops changing its position, indicating that the bulk value of the HOMO is reproduced in the center of the layer.

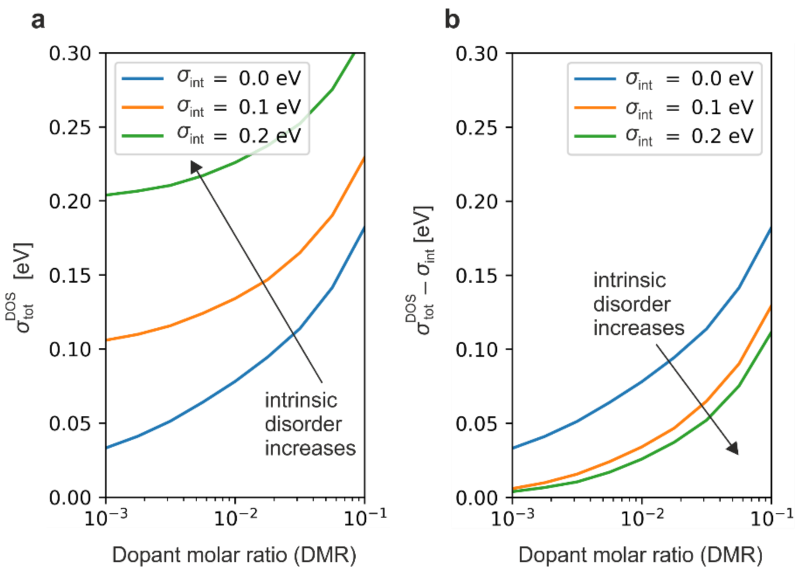

Supplementary Figure 7. a, Standard deviation of the HOMO and LUMO⁺ distribution (total disorder) $\sigma_{\mathrm{tot}}^{\mathrm{DOS}}$ of the of host molecules in the doped material as a function of the DMR for materials with various intrinsic disorder, σ_{int}. **b**, Excess disorder due to doping, $\sigma_{\mathrm{tot}}^{\mathrm{DOS}} - \sigma_{\mathrm{int}}$.

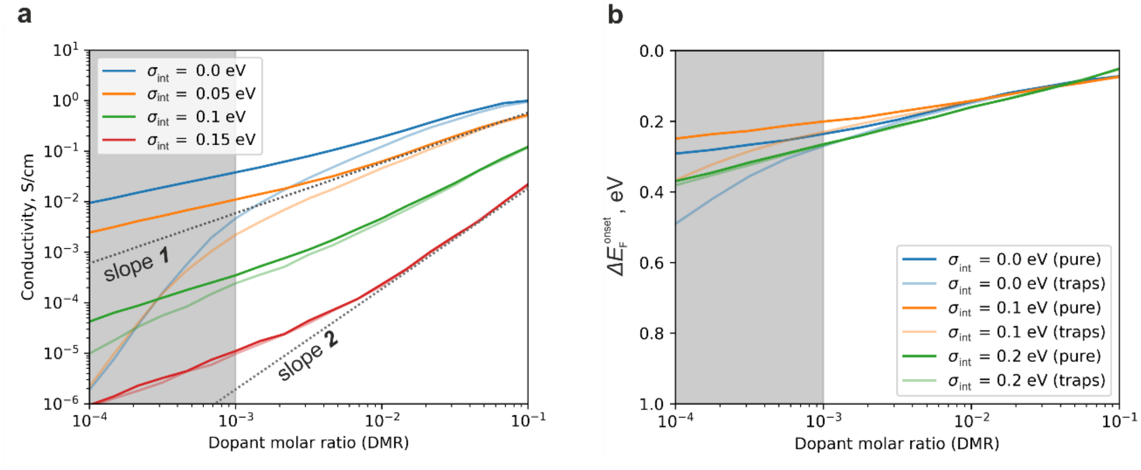

Supplementary Figure 8. Simulation of the doped organic semiconductor with traps. a, Conductivity vs. DMR. Solid and semi-transparent lines denote materials without and with traps, respectively. **b**, Fermi level shift w.r.t. HOMO onset ($\Delta E_{\mathrm{F}}^{\mathrm{onset}}$) vs. DMR. The effect of the trap states is noticeable mainly at ultralow doping concentration (filled in grey). At moderate doping levels, conductivity and Fermi level shift weakly depends on the presence or absence of traps. Simulations are done for various intrinsic material disorder (σ_{int}). Supplementary Note 7 provides details of the trap level.

Supplementary Notes

Supplementary Note 1: Polaron dynamics in doped organic semiconductors

First, we consider factors that determine ionization of the dopant. We start with the disorder-free doped organic semiconductor that consists of the host molecules (H) and dopant molecules (D). In this scenario, all host (dopant) molecules have the same electron affinity EA_h (EA_d) and ionization energy IP_h (IP_d) such that $IP_h - EA_d$ is small in comparison with the gap. An adjacent pair of D and H molecules can be found either in a neutral or ionized microstate (see **Supplementary Figure 1**) according to the local equilibrium of the reaction $\mathrm{H} + \mathrm{D} \leftrightarrows \mathrm{H}^+ + \mathrm{D}^-$. Neglecting electrostatic interaction with other molecules, the energy difference between these two microstates would be:

$$\Delta E = -\Delta E_{\mathrm{off}} + V_{\mathrm{C}}$$

where $\Delta E_{\mathrm{off}} = \mathrm{EA_d} - \mathrm{IP_h}$ is the molecule specific frontier orbital energy difference, and $V_{\mathrm{C}} = -\mathrm{e}^2/4\pi\epsilon\epsilon_0 a$ is the electrostatic interaction energy between the two molecules with a being the host-dopant separation, ϵ electric constant, ϵ_0 relative material permittivity and e elementary charge. Because this energy is always negative, charge separation can even occur if $\mathrm{EA_D} < \mathrm{IP_H}$.

In organic thin films and devices, the electrostatic interaction between the ionized pair (state "I") and positive (α) and negative (β) charges in the environment cannot be neglected, which introduces a third term to ΔE_{ext}:

$$\Delta E_{\mathrm{ext}} = \frac{e^2}{4\pi\epsilon\epsilon_0}\left[\sum_{\alpha}(r_\alpha - r_{\mathrm{h}})^{-1} - (r_\alpha - r_{\mathrm{d}})^{-1} - \sum_{\beta}(r_\beta - r_{\mathrm{h}})^{-1} - (r_\beta - r_{\mathrm{d}})^{-1}\right]$$

$r_\alpha, r_\beta, r_{\mathrm{h}}, r_{\mathrm{d}}$ are the positions of positive and negative charges in the environment and the positions of the host and dopant molecule, respectively. Due to the random dopant distribution in an amorphous thin film, ΔE_{ext} behaves as a stochastic quantity that can be either positive or negative. Moreover, this quantity changes dynamically depending on the position of all charges during the simulation. Note that ΔE_{ext} is computed using Ewald summation[1] as described in reference[2].

Intrinsic energy disorder results from slightly different molecular conformations and the unique electrostatic environment of each molecule, which is inherent to all disordered organic semiconductors. To include this effect, we employ the standard model that IP of host and EA of dopant molecules are distributed according to Gaussian distributions with the expectation values IP_h and EA_d and standard deviation σ_{IP} and σ_{EA}. Therefore the energy difference between the neutral and ionized state has an additional time-dependent stochastic contribution $\Delta E_{\mathrm{int}}(t) = \delta_{\mathrm{IP}}(t) - \delta_{\mathrm{EA}}(t)$, where $\delta_{\mathrm{IP}}(t) = \mathrm{IP_h}(t) - \mathrm{IP_h}$ and $\delta_{\mathrm{EA}}(t) = \mathrm{EA_d}\,(t) - \mathrm{IP_d}$ are molecule and time-dependent quantities, which characterize deviations of the IP and EA levels of a given pair of molecules from their expectation values.

Including all these contributions, the central equation of the dopant ionization process takes on a form:

$$\Delta E_{\mathrm{ion}} = \Delta E_{\mathrm{det}} + \Delta E_{\mathrm{int}} = -\Delta E_{\mathrm{off}} + V_{\mathrm{C}} + \Delta E_{\mathrm{ext}} + \Delta E_{\mathrm{int}} \pm eEr_{ji} \quad (1)$$

Supplementary Equation 1 shows that the dopant ionization energy is defined by the following contributions: energy difference between $\mathrm{IP_h}$ and $\mathrm{EA_d}$, host-dopant Coulomb interaction (always negative, for a dielectric permittivity of $\epsilon = 4$ and a host-dopant distance of 1 nm equal to 0.36 eV), intrinsic disorder (positive or negative), (doping-induced) environmental disorder (positive or negative), the local potential due to the applied electric field $\boldsymbol{E}$ (E and r_{ji} are vector quantities; signs "+" and "–" are for electrons and holes, respectively). In spite of their simplicity, the last two or even three terms of Supplementary Equation 1 to date not been considered in explicit mesoscopic models, mainly because of the associated numerical complexity. The hopping process between two host molecules and injection from electrodes if any is described with similar rates.

We note that IP and EA levels computed with Supplementary Equation 1 can only be compared directly to measurements of bulk materials and not gas or liquid phase measurements due to environment specific shifts of the energy levels[3,4].

Supplementary Note 2: Microscopic processes

The following kinds of microscopic processes are considered: hopping of holes/electrons; dopant ionization/neutralization and electrons/holes injection/ejection.

Hopping of holes and electrons. It is assumed that molecules can be in the neutral or first oxidation state. If a molecule is positively charged, this is interpreted as a missing electron (*hole*) in the HOMO; if it is negatively charged, it is interpreted as an additional *electron* in the LUMO. Changing of the oxidation state of the molecule due to charge transfer (for example in the reaction between two host molecules):

$$\mathrm{H^{+}H \rightarrow HH^{+}}$$

is interpreted as a *hole hop*. The hopping rate of an electron/hole from site i to j, W_{ij} is described by the Miller-Abraham (MA) expression[5]:

$$W_{ij} = \nu_0 \exp\left(-\frac{2r_{ij}}{b}\right) \exp\left[-\frac{|E_j - E_i| + (E_j - E_i)}{2k_{\mathrm{B}}T}\right] \quad (2)$$

where E_j and E_i energies of the final and initial state, ν_0 is the attempt-to-escape frequency, b is the localization radius of a charge carrier, k_{B} is Boltzmann constant, T is the temperature. The usage of MA expression in this work has been preferred to the Marcus hopping rate[6] to avoid one more free parameter (reorganization energy), which is not important to study the *general* doping-induced phenomena. Apart of this, it has been shown that both methods lead to similar results in bulk material simulations[7].

Corresponding energy difference is described by the last three terms of Supplementary Equation 1**.**
The energy difference for the hopping process of holes or electrons corresponds to the last three terms of Supplementary Equation 1:

$$\Delta E_{ji} = \Delta \mathrm{E}_{ji}^{\mathrm{ext}} + \Delta \mathrm{E}_{ji}^{\mathrm{int}} \pm eEr_{ji}, \quad (3)$$

where $r_{ji} = r_j - r_i$ is a hopping vector, where $\Delta \mathrm{E}_{ji}^{\mathrm{ext}}$ and $\Delta \mathrm{E}_{ji}^{\mathrm{int}}$ are differences in the energy of the site i and j due to extrinsic and intrinsic disorder.

Dopants activation. Hole dopant is by definition the molecule, whose EA is close to the IP of the host material (in terms of the molecular orbitals, $\mathrm{HOMO_h} \approx \mathrm{LUMO_d}$). To enable dopant activation (ionization of the dopant and generation of the hole on the host material) we allow the redox process between the host H and the dopant D to occur:

$$\mathrm{H} + \mathrm{D} \rightarrow \mathrm{H}^{+} + \mathrm{D}^{-}.$$

The generated charge in the host is now treated as a hole. The reciprocal process, a dopant deactivation, is also allowed. The rate of the dopant ionization (or activation) is also described by the MA expression (Supplementary Equation 2), where i and j are now states before and after dopant activation, r_{ij} is the dopant-host distance. It must be noted that the energy E_j of the ionized dopant-host pair is reduced due to attractive Coulomb energy of the generated charge pair.
For each kMC simulation performed in this work, we compute the time-averaged fraction of the ionized dopants η as:

$$\eta = \frac{1}{\tau_\Sigma} \sum_i N_\mathrm{d}^{-}(\tau_i)/N_\mathrm{d}$$

where τ_i is a dwelling time of the i-th microstate, $\tau_\Sigma = \sum_i \tau_i$; N_d and N_d^{-} are number of all and ionized dopants. The averaging is performed over the last 50% of the kMC trajectory.
In this manuscript, we have simulated a very strong dopant with EA higher than the host IP by 1.5 eV. Therefore, the computed value of η is 1 within a machine accuracy. This makes the molar ratio of ionized dopant defined as:

$$\mathrm{DMR}^{-} = N_\mathrm{d}^{-}/N_\mathrm{h},$$

(with N_d^{-} and N_h being number of ionized dopants and host molecules) equal to the dopant molar ratio (DMR) used overall in the paper and defined as:

$$\mathrm{DMR} = N_\mathrm{h}/N_\mathrm{d}.$$

Injection/ejection from electrodes. Electron/hole injection can be considered as following reactions (e^{-} stands for an electron):

$$\mathrm{H} + e^{-} \rightarrow \mathrm{H}^{-};$$
$$\mathrm{H} \rightarrow \mathrm{H}^{+} + e^{-}.$$

Injection rate is heuristically described by an Arrhenius-like equation:

$$W_{\mathrm{inj}} = \nu_0 exp(-r/d) exp(-\Delta E/k_{\mathrm{B}}T), \quad (4)$$

where ν_0 is an effective metal-organic coupling analogously to the attempt to jump frequency in the MA expression, r is the distance between the metal surface and the given molecule, d is an empirical parameter that accounts for the distance dependence of the metal-organic coupling.

For electron/hole injection, $\Delta E_{\mathrm{el}}/\Delta E_{\mathrm{hole}}$ are defined as

$$\Delta E_{\mathrm{el}} = (\mathrm{LUMO_h} - E_{\mathrm{F}}) - dF,$$

$$\Delta E_{\mathrm{hole}} = (E_{\mathrm{F}} - \mathrm{HOMO_h}) - dF,$$

where dF is the interaction energy between a charge and its mirror charge.

Supplementary Note 3: Extraction of the HOMO and LUMO⁺ distribution

The distributions of the HOMO and $\mathrm{LUMO^+}$ levels of the host molecules have been computed from kMC trajectory "measuring" site energies of neutral hosts as $^{-}$IP and site energies of ionized hosts as $^{-}\mathrm{EA^+}$. The resulting DOS is the sum of these two quantities. Note that hereinafter IP, EA, HOMO and $\mathrm{LUMO^+}$ though used without subscript "h" relate to host molecules.

In more details, at each i-th frame of the kMC trajectory we compute a binned distribution of the IP of the hosts, which are neutral at the i-th frame, $\mathrm{IP}_i(E)$, and IP of the hosts, which are positively charged at the i-th frame, $\mathrm{EA}_i^+(E)$, as well as the dwelling time τ_i of the system at the i-th frame. Then, we find the time-average of these distributions:

$\mathrm{IP}(E) = \frac{1}{\tau_\Sigma}\sum_i \tau_i \mathrm{IP}_i(E)$,

$\mathrm{EA^+}(E) = \frac{1}{\tau_\Sigma}\sum_i \tau_i \mathrm{EA}_i^+(E)$,

where $\tau_\Sigma = \sum_i \tau_i$.

Then, $\mathrm{HOMO}(E) = -\mathrm{IP}(E)$ and $\mathrm{LUMO^+}(E) = -\mathrm{EA^+}(E)$, which are what we call the density of the occupied and unoccupied states.

We note that this definition of the density of states takes into account that the energy of a given particle depends on the position of other $N-1$ particles with N being the total number of particles. To our understanding, this is the maximum information we can retrieve from the kMC trajectory to DOS. To our knowledge, although the density of states of doped

organic semiconductors has been recently reported[8], this type of the DOS averaging has never been done before. This limited the insights gained from kMC simulations, in particular about reliable information about the position of the doping-induced trap states and the DOS modification, which is essential to understand what governs the doping efficiency.

There is another possible way that one can use to compute DOS from the kMC trajectory. In this approach, one first computes time-averaged charge density at each site during some number of iterations (and thus, time-average external potential on each host site). After that, one computes the distribution of IP and EA^+ of the sites subjected to this average potential. In this case, valuable information about the correlations contained in the kMC trajectory is lost. Such a method is essentially a mean-field method. It is likely that in already mentioned work[8] authors used this "mean-field" method, as they observe no signs of EA^+ ($LUMO^+$).

We have implemented this latter method (referred to as "mean-field") and compared it to the one used throughout this work (referred to as "many-body"). The results are shown in **Supplementary Figure 2** for low (a) and high (b) dopant concentration. One can conclude that the $LUMO^+$ states are indeed missing. At low dopant concentration, HOMO distribution computed by "many-body" method and the total DOS computed by "mean-field" method are almost the same. At higher dopant concentration, however, even this fails: the "mean-field" DOS is shifted towards higher energies. Such "mean-field" method obviously prevent reasonable interpretation of the doping in organic semiconductors, which was fixed by our many-body approach.

Supplementary Note 4: Extraction of the bulk Fermi level shift

In case of the "bulk semiconductor"

The Fermi level E_F is extracted from kMC simulations of the "bulk" system *a posteriori* via the charge neutrality equation:

$$\mathrm{DMR}^- = \int \mathrm{DOS}(E)(1 - f_F(E, E_F))\, dE. \tag{5}$$

Here $\mathrm{DOS}(E)$ is the density of states (namely the number of host states per molecule of a material per energy unit) and $f_F(E, E_F)$ is the Fermi-Dirac distribution function. Polarons in the organic semiconductors are assumed to obey Fermi statistics.

The left part of this equation, DMR^- is the molar ratio of ionized dopants and the right part is the molar ratio of positively charged host molecules. As far as the material is neutral, the Fermi level can be found that turns this equation into equality. For the energetics of hosts and dopants used in this manuscript, all dopants are ionized and in Supplementary Equation 5: $\mathrm{DMR}^- = \mathrm{DMR}$.

As mentioned in the manuscript, the Fermi level is always the intersection energy of LUMO and HOMO associated DOS. This is because holes associated with LUMO distribution and electrons associated with HOMO distribution obey Fermi statistics, in which the Fermi level

corresponds to the energy where the state (if exists) is occupied with 50% probability. Therefore, the density of $LUMO^+$ and HOMO states is always the same at this energy. Supplementary Figure 3 exemplifies this for the case of the bulk material with intrinsic disorder 0.15 eV. Here, we plot HOMO and $LUMO^+$ distributions for eleven dopant molar ratios between 10^{-3} and 0.4 (dopant molar ratios are logarithmically spaced). The cross of the Fermi level as determined from the Supplementary Equation 5 where the HOMO *distribution* is plotted with a thick black point. As we can see, the Fermi level always crosses the HOMO distribution at the very same point where the LUMO distribution crosses the HOMO distribution, so that the Fermi level always hits the HOMO/$LUMO^+$ intersection.

Note that in this work we have computed the position of the $LUMO^+$/HOMO distribution with respect to the Fermi level using two independent methods. The first method is based on the charge neutrality equation (Supplementary Equation 5). The second method is based on the equilibration with the fixed chemical potential of electrodes (see below).

Although the second method does not require explicit postulation that the particles are Fermi distributed, both provide the same results and put the Fermi level exactly at the HOMO/LUMO intersection, which means that holes obey Fermi statistics.

In case of "single layer devices"

We simulate the "single layer device" system with initial energy levels as shown in **Supplementary Figure 4a.** To obtain a Fermi level of the *bulk* semiconductor, we use the following system specifications: the length of the system for each dopant concentration is chosen in a way that the space charge region is significantly shorter than the half of the layer thickness. This ensures that the properties in the center of the layer are the same as in a bulk semiconductor; to efficiently reach convergence of the charge distribution, hopping from the electrodes to each site of the left/right half of the organic system is allowed. The parameter d in Supplementary Equation 4 is assumed to be infinite, which means that each molecule of the semiconductor is connected to electrodes with the same electronic coupling strength. This influences the time scale of the kMC simulation but fulfills detailed balance and thus does not change the equilibrium distribution. In other words, each material site is connected directly to the electron reservoir (electrodes), and thus equilibration is achieved in the fastest possible way. In practice, all simulated systems reach equilibrium after 10^5 kMC steps that has been shown by longer simulations of 10^6 and 10^7 steps. The DOS results presented in the paper were time averaged over the10^3 last steps of a kMC trajectory of 10^6 steps.

When extracting Fermi level in the centre of organic *thin films* shorter than double the width of the depletion layer (this quantity has a physical sense of a "hole injection barrier"), the thickness of the layer is set to the value we are interested in (**Supplementary Note 5** provides more details). The rest of the described procedure holds.

Supplementary Figure 4b shows the DOS distribution along the single-layer device and the place where the distance between the Fermi level and HOMO offset/peak is measured.

Supplementary Figure 4c shows the cut of the DOS (from **Supplementary Figure 4b**) in the center of the layer.

Supplementary Note 5: The Fermi level shift in the thin doped layers

Supplementary Figure 5 shows the Fermi level shift with respect to the peak of the host HOMO level in the center of the simulating system "single layer device" as a function of the dopant molar ratio. As the simulating system becomes thicker, the position of the "turning point" between the steep and flat regions shifts towards smaller molar ratios, demonstrating that the origin of the steep region in the dependence of the Fermi level vs. dopant molar ratio is interface, not a bulk effect.

If the thickness of the simulated doped layer is smaller than the double thickness of the space charge region, then in the center of the layer we will have partially or fully depleted region and in this case the Fermi level shift $\Delta E_{\mathrm{F}}^{\mathrm{peak}}$ in the center of the system will be different from its value at an infinite distance from the metal surface (bulk value).

Supplementary Figure 6 shows the HOMO peak as a function of the distance for different thickness of the layer ranging from 10 to 50 nm. For small thickness, the HOMO peak in the center of the simulated layer may look flat, although the whole organic film is depleted. Only for the thickness of about 40...50 nm, the HOMO in the center of the system stops changing its position, indicating that the bulk value of the HOMO is reproduced in the center of the layer.

Supplementary Note 6: Disorder correlation for the occupied and free states

Our conclusions about the effect of the disorder compensation remains the same if we determine the total disorder of the material as the standard deviation of the distribution of the states, where holes can hope to (HOMO distribution), but both states filled by holes (distribution of LUMO^{+}) and the HOMO distribution, which we refer to as $\sigma_{\mathrm{tot}}^{\mathrm{DOS}}$. **Supplementary Figure 7** shows the same plots as in **Figure 5a,b** of a paper, but using the mentioned definition of the total material disorder.
Supplementary Figure 7a shows the total disorder of the full DOS, which is always larger for materials with higher intrinsic disorder. **Supplementary Figure 7b** that shows the excess disorder due to doping illustrates that in materials with higher intrinsic disorder, additional disorder defined as $\sigma_{\mathrm{tot}}^{\mathrm{DOS}} - \sigma_{\mathrm{int}}$ is much lower than in materials with low intrinsic disorder.

Supplementary Note 7: Effect of the trap states filling

In the ultralow doping regime, realistic semiconductors show another important effect, the passivation of traps[9]. Different trap states concentration have been reported ranging from 10^{16} cm^{-3} in pentacene[10] to 10^{18}... 10^{19} in C_{60}. These effects are observed as the prompt increase of the conductivity upon doping in the ultralow doping regime, normally below DMR < 10^{-3} as documented in the literature[9,11,12]. In order to show the applicability and the relevance of the disorder compensation effects to realistic organic semiconductors, we made a comparison of a pure materials simulated in the manuscript and the same materials, but with traps.

Supplementary Figure 8 shows the effect of the traps on the conductivity and the Fermi level position. We have added exponentially distributed trap level with parameters taken from the reference[9] (molar ratio of traps is 0.007) to the doped materials we have simulated in **Figure 4**. We have also extended and shifted the interval of the simulated dopant molar ratios down to DMR = 10^{-4} to better see the effect of traps passivation (this additional interval of DMR is filled in grey in **Supplementary Figure 8**). Conductivity of the materials with and without traps are plotted in the same color, but for materials with traps lines are semi-transparent. As reported in the literature and as expected from traps concentration, the main effect of the trap states vanishes shortly after dopant molar ratio exceeds 10^{-3}. After that, at moderate doping concentrations, conductivity increases either linearly or superlinearly depending on the intrinsic material disorder, which is explained in this work by means of the disorder compensation effect.

The slope of the Fermi level shift upon doping slightly increases mainly below DMR = 10^{-3}, but for bulk samples it is still 10 times less than 1 eV/dec. To explain UPS measurements that reports such a prompt (1 eV/dec) Fermi level shift, one needs to involve effects due to finite layer thickness as described above and in the manuscript.

It is noticeable that highly disordered materials are less sensitive to the traps that we have introduced (for example, the conductivity of the material with intrinsic disorder 0.15 eV doesn't visually change upon introducing traps, **Supplementary Figure 8a**). This is because exponentially distributed shallow traps, which we used in this case, are largely overlapped with the tail of the HOMO states, if HOMO intrinsic disorder is high. Thus, in highly disordered materials, relevant traps must be deeper to cause pronounced trap-filling effects.

Supplementary References

1. Ewald, P. P. Die Berechnung optischer und elektrostatischer Gitterpotentiale. *Ann. Phys.* **369**, 253–287 (1921).
2. Casalegno, M., Raos, G. & Po, R. Methodological assessment of kinetic Monte Carlo simulations of organic photovoltaic devices: The treatment of electrostatic interactions. *J. Chem. Phys.* **132**, 094705 (2010).
3. Schneider, T. *et al.* p-Doping of polystyrene polymers with attached functional side-groups from solution. *J Mater Chem C* **5**, 770–776 (2017).
4. Liu, G. *et al.* Mixed matrix formulations with MOF molecular sieving for key energy-intensive separations. *Nat. Mater.* **17**, 283–289 (2018).
5. Miller, A. & Abrahams, E. Impurity Conduction at Low Concentrations. *Phys. Rev.* **120**, 745–755 (1960).
6. Marcus, R. A. Electron transfer reactions in chemistry. Theory and experiment. *Rev. Mod. Phys.* **65**, 599 (1993).
7. de Vries, X., Friederich, P., Wenzel, W., Coehoorn, R. & Bobbert, P. A. Full quantum treatment of charge dynamics in amorphous molecular semiconductors. *Phys. Rev. B* **97**, 075203 (2018).
8. Zuo, G., Abdalla, H. & Kemerink, M. Impact of doping on the density of states and the mobility in organic semiconductors. *Phys. Rev. B* **93**, 235203 (2016).
9. Olthof, S. *et al.* Ultralow Doping in Organic Semiconductors: Evidence of Trap Filling. *Phys. Rev. Lett.* **109**, 176601 (2012).
10. Pahner, P. *et al.* Pentacene Schottky diodes studied by impedance spectroscopy: Doping

properties and trap response. *Phys. Rev. B* **88**, 195205 (2013).

11. Zhang, Y., de Boer, B. & Blom, P. W. M. Trap-free electron transport in poly(p - phenylene vinylene) by deactivation of traps with n -type doping. *Phys. Rev. B* **81**, 085201 (2010).
12. Tietze, M. L., Pahner, P., Schmidt, K., Leo, K. & Lüssem, B. Doped Organic Semiconductors: Trap-Filling, Impurity Saturation, and Reserve Regimes. *Adv. Funct. Mater.* **25**, 2701–2707 (2015).
